# Photoacoustic pump-probe tomography of fluorophores *in vivo* using interleaved image acquisition for motion suppression

**DOI:** 10.1038/srep40496

**Published:** 2017-01-16

**Authors:** Julia Märk, Asja Wagener, Edward Zhang, Jan Laufer

**Affiliations:** 1 Institut für Optik und Atomare Physik, Technische Universität Berlin, Hardenbergstraße 36A, 10623 Berlin, Germany; 2 Medizinische Klinik mit Schwerpunkt Hepatologie und Gastroenterologie, Charité – Universitätsmedizin Berlin, Campus Virchow-Klinikum, Augustenburger Platz 1, 13353 Berlin, Germany; 3 Department of Medical Physics and Biomedical Engineering, University College London, Gower Street, London WC1E 6BT, UK; 4 Institut für Radiologie, Charité – Universitätsmedizin Berlin, Charitéplatz 1, 10117 Berlin, Germany

## Abstract

In fluorophores, the excited state lifetime can be modulated using pump-probe excitation. By generating photoacoustic (PA) signals using simultaneous and time-delayed pump and probe excitation pulses at fluences below the maximum permissible exposure, a modulation of the signal amplitude is observed in fluorophores but not in endogenous chromophores. This provides a highly specific contrast mechanism that can be used to recover the location of the fluorophore using difference imaging. The practical challenges in applying this method to *in vivo* PA tomography include the typically low concentrations of fluorescent contrast agents, and tissue motion. The former results in smaller PA signal amplitudes compared to those measured in blood, while the latter gives rise to difference image artefacts that compromise the unambiguous and potentially noise-limited detection of fluorescent contrast agents. To address this limitation, a method based on interleaved pump-probe image acquisition was developed. It relies on fast switching between simultaneous and time-delayed pump-probe excitation to acquire PA difference signals in quick succession, and to minimise the effects of tissue motion. The feasibility of this method is demonstrated in tissue phantoms and in initial experiments *in vivo*.

The absorption-based contrast of photoacoustic (PA) tomography has been shown to provide high resolution *in vivo* images of the vasculature^1^,^2^, exogenous contrast agents^3^,^4^,^5^,^6^, and genetically expressed proteins^7^,^8^,^9^,^10^ and pigments^11^ in deep tissue. Fluorophores have been used as exogenous contrast agents in proof-of-principle studies to demonstrate their detection *in vivo* at greater depths and with higher spatial resolution than that provided by purely optical modalities^12^. A field of new potential applications has also been opened by the development of genetically expressed fluorescent proteins that absorb in the near-infrared spectral region where the optical tissue penetration depth is greatest^13^,^14^,^15^,^16^. However, the unambiguous detection of fluorophores against the overwhelming background absorption by endogenous chromophores remains challenging. The most widely used approach is based on multiwavelength imaging and the application of some form of spectral unmixing, such as model-based inversion schemes^17^,^18^,^19^. These methods aim to exploit the differences in the specific absorption spectra of the tissue chromophores to recover their spatial distribution, which would in turn enable functional and molecular imaging at depths beyond the ballistic regime^20^. They need to account accurately for the wavelength dependence of the fluence inside the illuminated tissue and are therefore computationally expensive. In addition, they rely upon prior information, such as the absorption and PA spectra of all chromophores, which are assumed to be constant during the process of PA signal generation. In fluorophores, this assumption is compromised if the excited state lifetime^21^ is comparable to the duration of the excitation pulse. Under these conditions, it has been shown that the absorption and the thermalisation are fluence dependent and deviate from those known *a priori*^21^,^22^.

Recently, an experimental method was demonstrated that has the potential to overcome these limitations^23^. It is based on PA pump-probe excitation which is used to modulate the comparatively long excited state lifetimes of fluorophores. The electronic and vibrational transitions in a fluorophore during pump-probe excitation are illustrated in Fig. 1(a). Following the absorption of a pump photon, the electronically and vibrationally excited fluorescent molecule first relaxes to the lowest vibrational level of the excited electronic state, a process that occurs on the order of ps^24^. It then returns to the electronic ground state via either the spontaneous emission of a photon, i.e. fluorescence, or non-radiative processes, such as vibrational decay, internal conversion or inelastic collisions. The different relaxation processes are competitive and the return to the ground state can be comparatively slow, occurring on time scales of several hundred ps to several ns^25^,^26^. Since only non-radiative relaxations contribute to the PA signal generation, the effects of (i) fluorescence emission and (ii) ground state depopulation during the ns excitation pulse^21^,^27^ combine to reduce the PA amplitude measured in fluorophores compared to that observed in non-fluorescent chromophores of identical absorption. Non-fluorescent chromophores, such as haemoglobin, water and lipids, exhibit short (ps) excited state lifetimes which result in fast vibrational relaxation to the ground state, and therefore the complete thermalisation of the optical energy of the pump photon. The excited state lifetime of fluorophores can be modulated using pump and probe excitation pulses. By using a probe pulse with a wavelength that corresponds to the fluorescence spectrum, stimulated emission (SE)^28^,^29^ is induced, which accelerates the relaxation of the long-lived excited states to the ground state. This causes a change in the amount of thermalized energy, and hence the PA signal amplitude. The degree to which SE modulates the signal amplitude can be controlled by changing the time delay between the pump and probe pulses, i.e. for simultaneous pump and probe pulses the effect of SE is at a maximum while for time delays longer than the fluorescence lifetime of the fluorophore, *τ*_f_, it is negligible. By subtracting PA signals generated using simultaneous and time-delayed pump and probe pulses, a difference signal is obtained. This is illustrated in Fig. 1(b), which shows a cuvette-based measurement as an example. Pump-probe excitation offers a PA contrast mechanism that is specific to fluorophores since signals generated in non-fluorescent, endogenous chromophores are unaffected by the time delay between the pulses, and therefore cancel out to yield potentially noise-limited detection.

While pump-probe difference imaging was first demonstrated in tissue phantoms^30^, challenges to its *in vivo* application remain. Since PA signals generated *in vivo* in weakly accumulated fluorescent contrast agents are smaller than those measured in blood and since tissue motion due to breathing or pulsatile blood flow can give rise to significant artefacts in the difference images, the unambiguous detection of fluorophores can be compromised. This is particularly noticeable if sequential image acquisition is used, i.e. the successive capture of two image data sets at different pump-probe time delays from which a difference image is then obtained. The disadvantage of this approach is the high likelihood of motion artefacts, especially if the image acquisition time is comparatively long. To address this limitation, an interleaved pump-probe image acquisition method was developed to allow difference imaging with minimal motion artefacts. This method was validated by imaging fluorophores in a motion-mimicking tissue phantom, and applied to *in vivo* imaging in an initial preclinical study.

## Results

Pump and probe excitation pulses were provided by an OPO laser system. The pump wavelength was set to 680 nm to coincide with the absorption peak of a NIR fluorophore (Atto680, Atto-Tec GmbH, Germany) while the probe wavelength of 742 nm coincided with the fluorescence spectrum to induce SE. An all-optical PA scanner based on a planar Fabry-Perot polymer film ultrasound sensor^31^ was used to acquire PA data sets from which 2-D and 3-D tomographic images were obtained using a time-reversal image reconstruction algorithm^32^. To produce PA difference signals, a time delay between the pump and probe pulses was introduced. This allowed interleaved image acquisition which relied on (1) switching between fixed time delays of 0.0 ns and 15.0 ns, achieved using optical fibres of different length, at the pulse repetition frequency of the excitation laser (50 Hz), and (2) measuring the two successive PA signals at each raster scan position of the PA scanner. This yielded a difference signal in which tissue motion artefacts that occur over time scales longer than 20 ms are eliminated. The experimental setup (MATERIALS AND METHODS) also enabled sequential image acquisition, i.e. the successive measurement of two image data sets using simultaneous and time-delayed pump and probe pulses, to allow a direct comparison of the methods.

### Pump-probe imaging of fluorophores in a tissue phantom

**Figure 2:**
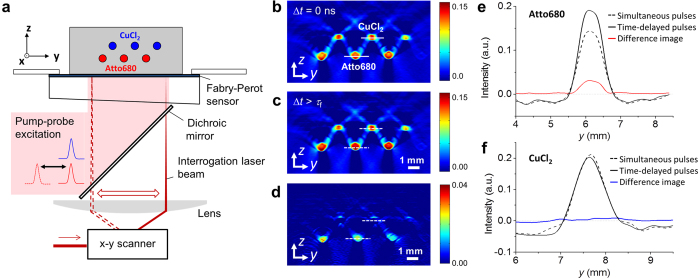
PA pump-probe imaging of a tissue phantom consisting of tubes filled with solutions of a fluorophore (Atto680) and a non-fluorescent absorber (CuCl_2_) immersed in a scattering lipid suspension. (**a**) Schematic of the experimental setup. 2-D cross sectional PA images were acquired using a Fabry-Perot-based PA scanner using simultaneous and time-delayed pump and probe pulses. (**b–d**) PA images acquired in a stationary phantom using sequential image acquisition using (**b**) Δ*t* = 0 ns and (**c**) Δ*t* > *τ*_f_, resulting in (**d**) a difference image. (**e,f**) Image intensity profiles corresponding to the dashed lines in (**b–d**) for tubes filled with (**e**) Atto680 and (**f**) CuCl_2_.

The phantom consisted of polymer tubes immersed in a scattering lipid suspension (Fig. 2(a)). The tubes were filled with solutions of either a NIR fluorescence dye (Atto680) or copper chloride (CuCl_2_), a non-fluorescent absorber mimicked endogenous tissue chromophores. Figure 2(b)–(f) illustrate the detection of fluorophores using pump-probe difference imaging. Figure 2(b) shows a 2-D cross sectional image of the phantom acquired using simultaneous pump-probe pulses while Fig. 2(c) shows that acquired using time-delayed pulses. Both images depict the location of all tubes irrespective of the type of absorber. Figure 2(d) shows the difference image obtained from Fig. 2(b) and (c). The location of the fluorophore-filled tubes is clearly visualised while the contrast corresponding to the non-fluorescent chromophore, i.e. CuCl_2_, is almost completely removed. Figure 2(e) and (f) show profiles of image intensities across tubes filled with Atto680 or copper chloride solutions as indicated by the dashed lines in Fig. 2(b)–(d). Figure 2(e) shows the difference in the PA image intensity that is produced in the fluorophore solution using pump-probe excitation with simultaneous and time-delayed pulses. Figure 2(f), by contrast, illustrates that pump-probe excitation does not modulate the image intensity measured in the CuCl_2_ solution, which results in almost complete cancellation of contrast in the difference image. Figure 2(b)–(f) illustrate that this method provides fluorophore-specific contrast whilst eliminating the background signals produced in non-fluorescent absorbers, such as endogenous tissue chromophores (blood, water and lipids). It should be noted that the image intensity measured in the fluorophore (Fig. 2(e)) is smaller for simultaneous compared to time-delayed pump and probe pulses. This is due to the specific experimental conditions of the tissue phantom experiment, i.e. a relatively high fluorophore concentration and a low excitation pulse fluence, which result in a low likelihood of repeated cycles of excitation and SE-induced relaxation per molecule during the pump and probe pulses. In the limit of a single excitation and SE-induced relaxation cycle, the generated fluorescence via SE reduces the number of vibrational transitions. This, in turn, leads to a reduction in thermalized energy and PA signal amplitude. However, for low concentrations, such as those typically found *in vivo*, pump-probe excitation is likely to result in multiple excitation-relaxation cycles per fluorophore molecule, which in turn increase the local temperature and pressure, and hence PA signal amplitude. The difference signal amplitude therefore depends on the ratio of fluorophore concentration and excitation fluence, which is discussed in more detail elsewhere^30^.

**Figure 3:**
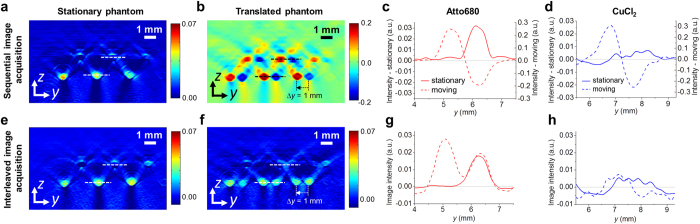
PA difference imaging of a tissue phantom using sequential (top row) and interleaved image acquisition (bottom row). (**a,b**) Cross sectional difference images of a phantom that was (**a**) stationary and (**b**) translated by 1 mm in *y* during sequential image acquisition. (**e**,**f**) Difference images of a (**a**) stationary and (**b**) translated phantom obtained using interleaved image acquisition. (**c**,**d**,**g**,**h**) Profiles of the image intensity along the dashed lines in (**a**,**b**,**e**,**f**) which correspond to the location of tubes filled with either the fluorophore (Atto680) or CuCl_2_ solution.

Difference images were obtained using sequential and interleaved image acquisition for two scenarios: (i) a stationary phantom and (ii) a phantom that was translated by approximately 1 mm in the *y* direction during the acquisition to mimic tissue motion. Figure 3(a) and (b) show cross-sectional difference images acquired using sequential image acquisition. Figure 3(a) shows the difference image of a stationary phantom in which the fluorophore-filled tubes are clearly visualized while the contrast generated by the background absorbers is minimized. Figure 3(b) shows the difference image of the translated phantom. It shows strong contrast for both types of absorber from which the start and end points of the translation can be estimated. Importantly, due to the positive and negative image intensities produced by all tubes, it is not possible to differentiate between the fluorophore and CuCl_2_ solutions. Figure 3(c) and (d) provide profiles of the image intensity along the solid and dashed lines shown in Fig. 3(a) and (b), corresponding to fluorophore and CuCl_2_ filled tubes. Figure 3(c) and (d) confirm that while sequential image acquisition provides fluorophore specific contrast and minimizes background signal in a stationary phantom, it is not suitable to difference imaging of moving targets. This is particularly evident in Fig. 3(d), which shows the difference image intensity profile across a CuCl_2_ filled tube. For a moving phantom, strong positive and negative difference contrast can be observed. This provides an illustration of the limitations of this method when applied *in vivo* where the movement of, for example, blood vessels could result in false positive detection of fluorophores.

This is in contrast to the results obtained using interleaved image acquisition shown in Fig. 3(e)–(h). Figure 3(e) shows the difference image of the stationary phantom, which shows the location of the fluorophore and is similar to Fig. 3(a) obtained using sequential acquisition. Figure 3(f) shows the difference image of the translated phantom. The locations of the fluorophore-filled tubes are clearly visible, showing the start and end points of the translation with positive image intensity. Importantly, the intensity corresponding to the CuCl_2_-filled tubes is strongly reduced. It is worth noting that interleaved image acquisition does not remove the effects of slow tissue motion, which occurs on times scales longer than the pulse repetition interval. Such comparatively slow movements will result in blurring of the difference image. This can be seen in Fig. 3(f), which shows the start and end points of the translation the fluorophore-filled tubes. For *in vivo* imaging of moving targets, such as fluorophore-labelled tissue regions in a small animal, the difference image would therefore show a blurred distribution of the contrast agent. The endogenous contrast is nevertheless almost completely removed, as illustrated in Fig. 3(f). An exception is a residual signal from a CuCl_2_ filled tube on the right hand side of the images in Fig. 3(e) and (f). This is most likely due to a difference in the fluence distribution caused by imperfect alignment of the optical fibres used to guide the probe pulses (see MATERIALS AND METHODS). Figure 3(g) and (h) show profiles of the image intensity corresponding to the dashed lines in Fig. 3(e) and (f). The profiles confirm that pump-probe excitation in combination with interleaved image acquisition provides fluorophore-specific contrast while strongly suppressing motion-induced background contrast that originates from non-fluorescent absorbers. The noise-equivalent concentrations estimated from the difference images of the stationary phantom are 5.4 μM and 4.3 μM for the sequential and interleaved image acquisition, respectively.

### PA pump-probe difference imaging of a NIR fluorophore *in vivo*

**Figure 4:**
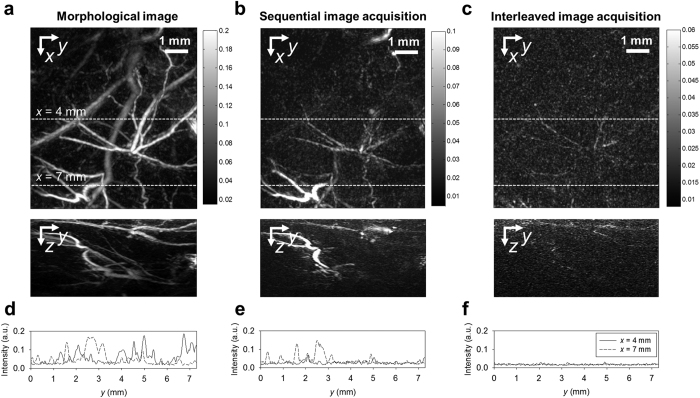
Comparison of the background suppression efficiency provided by sequential and interleaved image acquisition *in vivo: x-y* and *z-y* maximum intensity projections (MIP) of (**a**) the morphological PA image of the flank of a NMRI nude mouse showing the vasculature in the skin and underlying muscletissue, and the difference images acquired using (**b**) sequential image acquisition and (**c**) interleaved image acquisition. Profiles of the image intensity for *x* = 4 mm and 7 mm, i.e. along the dashed lines in the *x-y* MIPs in **(a–c**), are shown in (**d–f**).

The capabilities of sequential and interleaved image acquisition to detect fluorophores *in vivo* were tested by imaging a subcutaneous injection of a mixture of Atto680 and Matrigel in the flank of a NMRI nude mouse. 3-D image data sets were acquired using the PA small animal imaging system as described in MATERIALS AND METHODS. To compare the ability of the two methods to minimize motion artefacts, morphological and difference images of the flank were obtained using sequential and interleaved acquisition prior to injection. Figure 4(a) shows the *x-y* and *z-y* maximum intensity projections (MIP) of the 3-D image data set acquired using simultaneous pump and probe excitation pulses in which the vascular morphology is clearly visualized. Figure 4(d) shows image intensity profiles as a function of *y* for *x* = 4 mm and *x* = 7 mm, corresponding to the dashed lines in Fig. 4(a). Figure 4(b) shows the *x-y* and *z-y* MIPs of the difference image of the same region acquired using sequential image acquisition in which a number of blood vessels can be identified. Figure 4(e) again shows the intensity profile along the dashed lines in the *x-y* MIP (Fig. 4(b)). The intensity of the motion-induced artefacts in the difference image is comparable to that of the morphological image (Fig. 4(d)). This illustrates that small movements of the tissue during sequential image acquisition generate substantial artefacts. The results of the interleaved image acquisition are shown in Fig. 4(c) and (f). The *x-y* and *z-y* MIPs (Fig. 4(c)) show that the residual difference image intensities, which correspond to endogenous background absorbers are much lower than those shown in Fig. 4(a) and (b). The residual difference signals are likely due to tissue movement that occurs on time scales shorter than the pulse repetition interval. This is confirmed by the image intensity profiles for *x* = 4 mm and 7 mm (Fig. 4(f)), which show peak intensities that are an order of magnitude smaller than those in Fig. 4(d) and (e).

**Figure 5:**
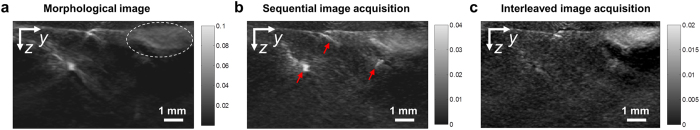
Detection of a NIR fluorophore *in vivo* using morphological and pump-probe difference imaging: (**a**) *z-y* MIP of the morphological image showing the region of Atto680 injection as indicated by the dashed line. (**b**) *z-y* MIP of the difference image acquired using sequential acquisition. Red arrows indicate residual motion artefacts due to moving blood vessels. (**c**) *z-y* MIP of the difference image acquired using interleaved image acquisition, which provides maximum contrast to the fluorophore.

After the injection of the Atto680 and Matrigel mixture into the flank of a NMRI nude mouse, morphological and pump-probe difference images were obtained. Figure 5(a) shows the *z-y* MIP of the morphological image in which blood vessels and the location of the fluorophore injection, as indicated by the dashed line, can be seen. The MIP of the corresponding difference image obtained using sequential image acquisition is shown in Fig. 5(b). Again, the location of fluorophore can be discerned but the image is also corrupted by difference contrast that stems from moving blood vessels (red arrows), which adversely affects the detection of the fluorophore. By contrast, Fig. 5(c) shows the difference image MIP obtained using interleaved acquisition in which the location of the fluorophore is detected with high contrast. The difference image nevertheless still shows weak, residual contrast from endogenous, non-fluorescent chromophores, which may stem from tissue movements that are faster than the pulse repetition interval of 20 ms.

**Figure 6:**
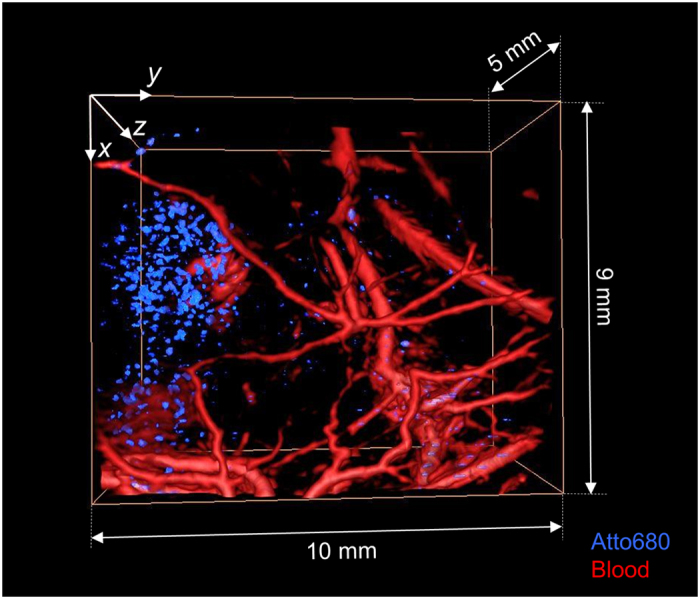
Volume-rendered, fused colour 3-D image of the vasculature in the flank of a NMRI nude mouse and the spatial distribution of a NIR fluorophore (Atto680) acquired *in vivo* using interleaved pump-probe difference imaging. The vasculature is shown in red and the fluorophore is shown in blue.

Figure 6 shows a volume-rendered, fused-colour 3-D image of the fluorophore injection site in the mouse flank obtained from the morphological and pump-probe difference image data sets. The blood vessel network and the fluorophore were visualized by thresholding the image data sets. The blood vessels are shown in red and the location of the fluorophore is shown in blue. The thresholded difference image intensity is more pronounced in the left half of the image which corresponds to the location where the Atto680 solution was injected. However, the difference image also shows contrast in other regions, such as in or around the blood vessels in the lower right hand corner, suggesting the presence of the fluorophore. While this cannot be ruled out entirely since a slow, gradual washout of the injected fluorophore was observed, it would seem an unlikely explanation since the duration of the experiments was minimized in order to avoid this effect. Again, tissue motion occurring within the pulse repetition interval of 20 ms may be a more likely reason.

## Discussion

This study has demonstrated that fluorophores can be detected *in vivo* using interleaved PA pump-probe tomography. While sequential image acquisition was shown to remove the overwhelming image contrast generated in endogenous tissue absorbers, its ability to detect fluorophores in the presence of motion was found to be limited. By minimizing the time between the acquisition of PA signals generated using simultaneous and time-delayed pump and probe pulses, interleaved image acquisition reduces tissue motion artefacts in pump-probe difference images with high efficiency. Interleaved PA pump-probe tomography of fluorophores combines a number of advantages. It provides fluorophore-specific contrast by suppressing the endogenous background signal and therefore offers near noise-limited sensitivity. It operates in tomography mode to allow deep tissue imaging and the fluences required are below the maximum permissible exposure (MPE) for skin, which is promising for potential clinical applications. The experimental approach is simple and can be implemented using a single OPO laser system. The advantages of interleaved PA pump-probe tomography are in contrast to approaches based on multispectral image acquisition and spectral unmixing, which require the acquisition of images at multiple excitation wavelengths, are computationally expensive and can be adversely affected by uncertainties in *a priori* information, such as the absorption and thermalisation exhibited by fluorophores during high peak power excitation pulses^21^. Furthermore, PA pump-probe difference imaging has been used to visualize differences in the excited state lifetime^33^ in phantoms. This may find applications *in vivo* where sensing of environmental parameters and multiplexed detection are of interest.

The method presented in this paper nevertheless has limitations that will need to be addressed if it is to become a practical tool for PA imaging of fluorophores. For example, tissue motion occurring on time scales shorter than the pulse repetition interval resulted in residual difference image contrast. This may be overcome by using, for example, two separate and independently triggered excitations lasers, which would provide minimal pulse repetition intervals and arbitrary pump-probe time delays. Variations in the probe fluence distribution, which were due to the use of two separate optical fibres to delay the probe pulse, also adversely affected the difference images by showing residual contrast from non-fluorescent absorbers. Since pump-probe difference imaging relies on constant fluence distributions, the probe beams need to be co-aligned with sufficient accuracy. The experimental setup used here was not optimal in this respect, which resulted in small fluence variations near the edges of the probe beam. Similarly, residual background contrast may also be attributed to fluence changes associated with the SE-induced modulation of Atto680 absorption. In order to enable this proof-of-principle study, the fluorophore concentrations used in the phantom and *in vivo* were significantly higher than those that may be found in tissue, for example, in studies where targeted contrast agents are administered systemically. Fluorophore photobleaching and washout may also need to be considered. While minor photobleaching was observed, its effects were minimized by using freshly prepared fluorophore solutions and by replacing the solutions before each phantom measurement. During the in vivo experiments, slow washout of the fluorophore due to tissue perfusion was observed because it was not targeted at specific cellular receptors. However, since the time scales of the washout were longer than typical image acquisition times, its effects can be considered negligible. The detection sensitivity of the method may be improved by employing highly parallelized detection and signal averaging to increase the signal-to-noise ratio. In addition, the creation of nanoparticles via encapsulation of fluorophores may also provide high local concentrations for robust detection.

In conclusion, interleaved PA pump-probe tomography has been shown to allow the detection of a fluorophore *in vivo* against the overwhelming background contrast of endogenous chromophores and in the presence of tissue motion. This method has the potential to enable PA imaging of exogenous fluorescent contrast agents or genetically expressed fluorescent proteins in deep tissue, which may find application in a wide range of preclinical and clinical studies.

## Materials and Methods

### Experimental Setup

**Figure 7:**
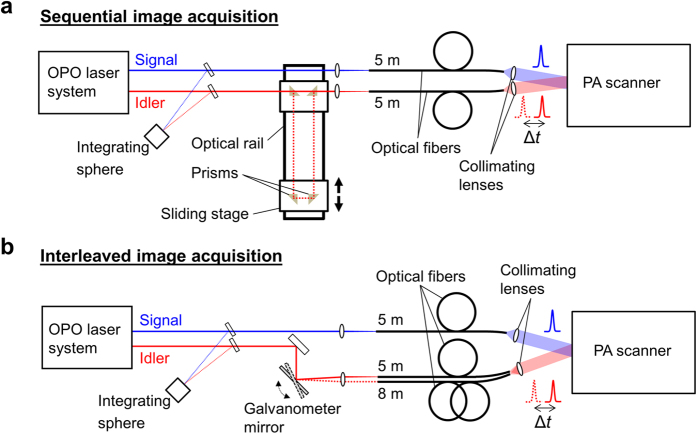
Schematics of the experimental setups for sequential and interleaved PA image acquisition using pump-probe excitation. (**a**) Sequential image acquisition relied on a free-space optical delay line to produce a time delay of up to 7.7 ns between the pump and probe pulses. (**b**) Interleaved image acquisition was achieved by measuring two signals generated using simultaneous and time-delayed pump and probe pulses at each raster scan position of the PA scanner. The probe beam was coupled into two optical fibres of different length to provide a time delay of 15 ns. A galvanometer mirror was used to switch the fibre coupling of the probe pulses at the PRF of the excitation laser (50 Hz).

The experimental setups for sequential and interleaved PA difference imaging are shown in Fig. 7. Pump and probe excitation pulses of 7 ns duration were provided by a wavelength tuneable OPO laser system (Quanta-Ray PRO-270-50, Newport Spectra Physics, USA and premiScan OPO, GWU, Germany) at a repetition frequency of 50 Hz. Small portions of the OPO output were directed to an integrating sphere where the pulse energy was measured using a wavelength calibrated photodiode and integrator, and the excitation wavelength using an USB spectrometer (Ocean Optics, USA). The pump and probe wavelengths were 680 nm and 742 nm, respectively.

The experimental setup for sequential image acquisition is shown in Fig. 7(a). For simultaneous pump and probe pulses, the signal and idler beams were coupled into fused silica multimode fibres of 1.5 mm core diameter and 5 m length. A time delay was introduced between the pump and probe pulses using an optical delay line, which consisted of prism reflectors mounted onto sliding stages and an optical rail. This provided a maximum time delay of Δ*t* = 7.7 ns, which was found to provide sufficient SE suppression for the fluorophore used in this study. The output of the fibres was collimated and directed to a PA scanner based on a planar Fabry-Perot polymer film sensor described in detail elsewhere^31^, where the co-aligned beams illuminated the target to generate PA waves as illustrated in Fig. 2(a). The beam diameters were approximately 2 cm (FWHM). The Fabry-Perot sensor consists of two dichroic mirrors of high transmittance between 590 nm and 1200 nm and high reflectance around 1550 nm, separated by a polymer film. Its optical transparency in the visible and near-infrared wavelength region allows the transmission of excitation laser pulses through the sensor and into the adjacent target to generate PA waves. The optical thickness of the etalon is modulated by the acoustic waves as they propagate through the sensor, causing a transient change in its reflectivity. By raster scanning a focussed 1550 nm CW laser beam (Tunics T100, Yenista, France) across the surface of the sensor and by recording the time-varying reflected intensity at each point using a photodiode, the spatial-temporal distribution of the incident PA waves are mapped in 2D. The photodiode output was recorded using a digitizer card (PCI-5124, National Instruments, USA). From this data, 3-D images can be obtained using image reconstruction algorithms. In this study, a Fabry-Perot sensor of 20 μm thickness was used, which provides a detection bandwidth of 39 MHz (−3 dB point). Sequential image acquisition involved the use of a fixed time delay between the pump and probe pulses, i.e. either Δ*t* = 0.0 ns or Δ*t* = 7.7 ns. For each Δ*t*, a 3-D PA image data set was recorded by acquiring PA signals in a 2-D raster-scan across the Fabry-Perot sensor.

The setup for interleaved image acquisition is shown in Fig. 7(b). The method relies on the acquisition of two PA signals using simultaneous and time-delayed pump and probe pulses at each raster scan position. Fast switching of the pump-probe time delay is achieved by deflecting the probe beam into optical fibres of either 5 m or 8 m length using a galvanometer mirror, resulting in time delays of Δ*t* = 0.0 ns or Δ*t* = 15.0 ns. The use of two separate fibres resulted in a 2mm offset of the signal and idler beam axes. PA image data sets were again acquired by raster scanning across the Fabry-Perot sensor.

Prior to image reconstruction, the PA signals were low pass filtered (*f*_c_ = 10 MHz) and 3-D images of the initial pressure distribution were reconstructed using a time-reversal-based algorithm^32^. A difference image was obtained by subtracting the images acquired using simultaneous and time-delayed pump-probe pulses.

### PA difference imaging in tissue phantoms

The phantom consisted of polymer tubes (600 μm inner dia., MORCAP, Paradigm Optics Inc., USA) filled with either a solution of a near-infrared fluorophore (Atto680, Atto-tec GmbH, Germany) in methanol or an aqueous solution of copper chloride (CuCl_2_), which mimicked endogenous absorbers, such as haemoglobin. The Atto680 solution had a concentration of 80 μM, which corresponds to an absorption coefficient of *μ*_a_ = 2.3 mm^−1^ at 680 nm. The fluorescence lifetime of Atto680 in methanol is 2.7 ns. The CuCl_2_ solution had a concentration of 0.9 M, i.e. *μ*_a_ = 2.5 mm^−1^ at 742 nm. The tubes were immersed in a scattering lipid suspension with a reduced scattering coefficient of *μ*_s_’ ~ 1 mm^−1^. The pump fluence was set to 2 mJ/cm^2^ and the probe fluence was set to 4.3 mJ/cm^2^. The phantom was placed onto the Fabry-Perot-based PA scanner. PA image data sets were acquired over a scan area of 5 mm × 16 mm using sequential and interleaved image acquisition. To mimic tissue motion, the phantom was translated by approximately 1 mm halfway during each image acquisition. To compensate for minor fluence changes, the intensity of the images was normalized to that measured in the tubes filled with Atto680 acquired using time-delayed pump and probe pulses. Subsequently, difference images were calculated by subtracting the images obtained using simultaneous and time-delayed pump-probe excitation.

### PA difference imaging *in vivo*

For *in vivo* experiments, at least 9-week-old female NMRI-Foxn1 nu/Foxn1 nu nude mice were used (Janvier Labs, France). All experiments were in accordance with the German Animal Welfare Legislation and were approved by the Landesamt für Gesundheit und Soziales Berlin (LaGeSo approval no. G0155/15). A nude mouse was anaesthetized using isoflurane and oxygen and placed onto the Fabry-Perot-based PA scanner. Aqueous gel provided acoustic coupling. The pump and probe fluences were 7 mJ/cm^2^ and 5 mJ/cm^2^, respectively, and therefore below the ANSI limit of maximum permissible exposure (MPE).

To compare the efficiency with which the background contrast is minimized, PA images of the blood vessels in the flank were acquired using sequential and interleaved image acquisition. Image data sets were obtained by recording PA signals (2000 points, sampling interval 5 ns) over a planar detection aperture of 8.0 mm × 6.5 mm with a step size of 100 μm for simultaneous and time-delayed pulses.

A volume of 150 μl of an aqueous solution of Atto680 mixed 1:1 with Matrigel (Corning Life Sciences, USA) with a fluorophore concentration of 100 μM was injected subcutaneously in the flank. Images of a tissue slice were recorded using sequential and interleaved image acquisition over an *x-y* detection aperture of 2.5 mm × 9.5 mm with step sizes of *dx* = 200 μm and *dy* = 100 μm, respectively. Increasing the step size in *x* minimized the image acquisition time, which in turn reduced potential errors due to fluorophore wash out or bleaching to enable a direct comparison of the difference images obtained using sequential and interleaved acquisition. A 3-D image of the fluorophore injection site in the flank was obtained using interleaved image acquisition over a detection aperture of 9 mm × 10 mm with a step size of 100 μm. With the exception of the last image data set, all PA signals were low pass filtered (*f*_c_ = 10 MHz) and images of the initial pressure distribution were calculated using a time-reversal algorithm. 3-D volume rendering, image segmentation and fused-colour visualisation was accomplished using Amira^34^.

## Additional Information

**How to cite this article:** Märk, J. *et al*. Photoacoustic pump-probe tomography of fluorophores *in vivo* using interleaved image acquisition for motion suppression. *Sci. Rep.* **7**, 40496; doi: 10.1038/srep40496 (2017).

**Publisher's note:** Springer Nature remains neutral with regard to jurisdictional claims in published maps and institutional affiliations.

## Figures and Tables

**Figure 1:**
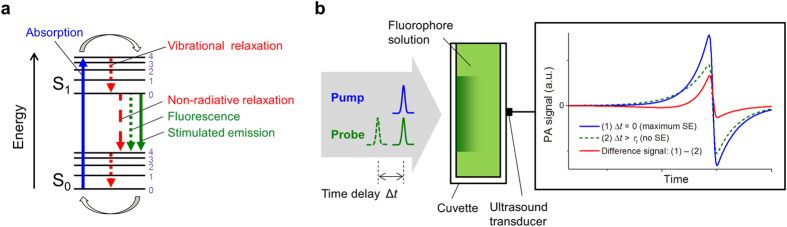
(**a**) Schematic of the electronic and vibrational transitions in a fluorophore during pump-probe excitation (S_0_ - electronic ground state, S_1_ - first excited electronic state). (**b**) Illustration of the changes in the PA signal amplitude measured in a fluorophore solution in a cuvette using pump-probe excitation. By changing the time delay between the pump and probe pulses, the PA signal amplitude is modulated, yielding a difference signal (*τ*_f_ - fluorescence lifetime).
